# Supplementary material for: A multisite validation of brain white matter pathways of resilience to chronic back pain
Source: eLife. 2024 Dec 24;13:RP96312. doi: 10.7554/eLife.96312 (PMC11668529; doi:10.7554/eLife.96312)
Supplement: Supplementary file 1. — A trained psychologist interviewed all participants to assess comorbid mental disorders using the German version of the Structured Clinical Interviews (SCID I) for the Diagnostic and Statistical Manual of Mental Disorders (DSM IV)(Wittchen, 1997). List of all reported diagnoses across groups is provided in Supplementary file 1—Table 1. [file elife-96312-supp1.docx]

| **Supplementary File 1—table 1 Comorbid mental disorders (Mannheim sample); diagnoses according to the Diagnostic and Statistical Manual of Mental Disorders IV (DSM IV)** | | | | |
| --- | --- | --- | --- | --- |
|  | **Code** | **Diagnosis** | **Acute** | **Remitted** |
| **SBP** | 296.26 | Major depressive disorder, single episode |  | 10 |
|  | 296.30/296.36 | Major depressive disorder, recurrent | 1 | 1 |
|  | 300.29 | Specific phobia | 1 |  |
|  | 300.30 | Obsessive-compulsive disorder |  | 1 |
|  | 305.xx | Abuse: Opioids/Amphetamine/Cannabis/ Sedative-, hypnotic-, or anxiolytic-related |  | 1 |
|  | 307.10 | Anorexia Nervosa |  | 2 |
|  | 307.51 | Bulimia Nervosa |  | 2 |
|  | 309.81 | Posttraumatic stress disorder |  | 2 |
| **CBP** | 296.26 | Major depressive disorder, single episode |  | 2 |
|  | 296.33 | Major depressive disorder, recurrent severe without psychotic features |  | 2 |
|  | 296.36 | Major depressive disorder, recurrent | 2 | 1 |
|  | 300.01 | Panic disorder, without agoraphobia | 2 |  |
|  | 300.22 | Agoraphobia without history of panic disorder |  | 1 |
|  | 303.90 | Dependence: Alcohol |  | 1 |
|  | 304.10 | Dependence: Sedative-, hypnotic-, or anxiolytic-related |  | 1 |
|  | 304.30 | Dependence: Cannabis |  | 1 |
|  | 305.xx | Abuse: Opioids/Amphetamine/Cannabis/ Sedative-, hypnotic-, or anxiolytic-related |  | 1 |
|  | 307.10 | Anorexia Nervosa | 1 |  |
|  | 307.51 | Bulimia Nervosa |  | 1 |
